# Reliability and validity of the German version of the University of Jyvaskyla Active Aging Scale (UJACAS-G)

**DOI:** 10.1186/s41687-024-00786-w

**Published:** 2024-09-10

**Authors:** Timo Hinrichs, Taina Rantanen, Erja Portegijs, Lukas Nebiker, Roland Rössler, Fabian Schwendinger, Arno Schmidt-Trucksäss, Ralf Roth

**Affiliations:** 1 Department of Sport, Exercise, and Health, University of Basel, Grosse Allee 6, Basel, 4052 Switzerland; 2 Faculty of Sport and Health Sciences and Gerontology Research Center, University of Jyväskylä, Jyväskylä, Finland; 3 Center for Human Movement Sciences, University of Groningen, University Medical Center Groningen, Groningen, The Netherlands; 4 Basel Mobility Center, University Department of Geriatric Medicine Felix Platter, University of Basel, Basel, Switzerland

**Keywords:** Aging, Geriatric assessment, Quality of life, Health status, Psychometrics

## Abstract

**Background:**

The University of Jyvaskyla Active Aging Scale (UJACAS) assesses active aging through willingness, ability, opportunity, and frequency of involvement in activities. Recognizing the lack of a German version, the Finnish original was translated (UJACAS-G). This study aimed: (1) to evaluate the test-retest reliability of UJACAS-G; and (2) to explore correlations with health-related parameters (concurrent validity).

**Methods:**

The study (test-retest design) targeted healthy older adults aged 65+. Reliability of UJACAS-G (total and subscores) was assessed using Bland-Altman analyses and Intraclass Correlation Coefficients (ICCs). Furthermore, correlations (Spearman’s rho) between UJACAS-G scores and physical function (walking speed, handgrip strength, balance, 6-minute walk distance), physical activity (International Physical Activity Questionnaire), life-space mobility (Life-Space Assessment), and health-related quality of life (Short Form-36 Health Survey) were calculated.

**Results:**

Bland-Altman analyses (*N* = 60; mean age 72.3, SD 5.9 years; 50% women) revealed mean differences close to zero and narrow limits of agreement for all scores (total score: mean difference −1.9; limits −31.7 to 27.9). The ability subscore showed clustering at its upper limit. ICC was 0.829 (95% CI 0.730 to 0.894) for the total score and ranged between 0.530 and 0.876 for subscores (all *p*-values < 0.001). The total score correlated with walking speed (rho = 0.345; *p* = 0.008), physical activity (rho = 0.279; *p* = 0.033) and mental health (rho = 0.329; *p* = 0.010).

**Conclusions:**

UJACAS-G is reliable for assessing active aging among German-speaking healthy older adults. A potential ‘ceiling effect’ regarding the ability subscore should be considered when applying UJACAS-G to well-functioning populations. Analyses of concurrent validity indicated only weak correlations with health-related parameters.

**Supplementary Information:**

The online version contains supplementary material available at 10.1186/s41687-024-00786-w.

## Background

The concept of active aging is crucial in addressing both the challenges and opportunities presented by an aging population. At the societal level, the policy framework developed by the World Health Organization (WHO)’s ‘Aging and Life Course’ programme in 2002 emphasizes optimizing health, participation, and security to enhance quality of life in older age [1]. Strategies derived from this framework are assessed using tools like the Active Aging Index [2], which evaluates societal indicators such as environmental support, societal participation, and employment opportunities. Yet, the WHO’s focus remains largely policy-oriented, lacking direct applicability to research on individual experiences of aging [3].

At the individual level, active aging encompasses older adults actively contributing to their own wellbeing by engaging in self-selected activities that align with their interests, abilities, and personal values [4–6]. Research highlighting the negative impact of chronic health conditions and functional limitations on aging experiences [7, 8] emphasizes the importance of considering individual health status when conceptualizing active aging. Stephens et al. [5] observed that perceptions of health and wellbeing may vary among older people based on their physical capacities. Building on these insights, Rantanen et al. [9] offered a nuanced, holistic definition of active aging as “the striving for elements of well-being through activities relating to a person’s goals, functional capacities, and opportunities.” This approach expands beyond societal measures, focusing on the resources individuals need to pursue what is meaningful to them.

The University of Jyvaskyla Active Aging Scale (UJACAS) [10], developed in alignment with this definition and inspired by the International Classification of Functioning, Disability, and Health (ICF)’s Activities and Participation chapter [11], assesses active aging through willingness, ability, opportunity, and frequency of involvement in various activities [10]. The scale’s novelty lies in its broad, inclusive approach, capturing diverse forms of activity suitable for individuals regardless of functional status. Aligning with the scale’s focus on capturing personal experiences and subjective perceptions of active aging, UJACAS has been designed as a self-report questionnaire, which may be administered online, in interviews, or as a paper and pencil assessment [10, 12].

The primary purpose of using the UJACAS is to provide a comprehensive measure of active aging at an individual level, offering insights into how older adults engage in meaningful activities that promote their well-being [10]. More specifically, the UJACAS enables the quantification of active aging as an entity that can vary from low to high. This quantification allows for the analysis of individual physical and mental characteristics, as well as environmental and social factors, as potential determinants or modifiers of active aging. Its application in cohort studies allows to assess whether different components of active aging have distinct predictors, how active aging changes with age, and whether active aging helps mitigate declines in well-being during periods of functional loss or disease. The UJACAS can also be used to monitor changes in active aging and to evaluate the effectiveness of interventions or technological solutions aimed at promoting active aging. Ultimately, the UJACAS can be utilized in implementation research and policy formulation, providing valuable insights for developing strategies to promote active aging at both individual and societal levels [10].

The UJACAS has demonstrated robust psychometric properties in various contexts [10, 12, 13]. The original Finnish version exhibited high test-retest reliability—with an intraclass correlation coefficient (ICC) of 0.915 for the total score—and has demonstrated validity through moderate correlation with the degree of activity and involvement in meaningful tasks assessed by an occupational therapist within a personal interview [10]. Existing adaptations of the UJACAS to other languages, so far Turkish [13] and Swedish [12], have also shown good psychometric properties.

The UJACAS has been shown to be feasible in large scale observational [9, 14, 15] and interventional studies [16]. In a population-based sample of older adults (*N* = 809) aged 75, 80 or 85, it has been shown that higher UJACAS scores were associated with higher quality of life [17]. It has also been found that having difficulties walking was associated with lower active aging scores [18], that psychological resilience had a protective effect on active aging [19], and that older men in senior houses had lower active aging scores compared to their community-dwelling counterparts [20].

Recognizing the lack of a German version of the UJACAS, our study translated the original Finnish version into German (UJACAS-G). The primary aim was to evaluate the test-retest reliability of UJACAS-G among healthy older adults. Additionally, to evaluate the concurrent validity, we explored correlations between UJACAS-G scores and various health-related parameters, including physical function, physical activity, life-space mobility, and health-related quality of life. These parameters were selected because they are well-established indicators of health and functional status in older adults and are relevant to the construct of active aging. We hypothesized that higher UJACAS-G scores would be associated with better physical function, higher levels of physical activity, greater life-space mobility, and higher quality of life.

## Methods

### Study design, target group and recruitment

This observational study was approved by the Ethics Committee Northwest and Central Switzerland (Reg.-No. 2021-01683). A test-retest design was used to assess reliability. Assessments took place at the study center (Department of Sport, Exercise and Health; University of Basel) at two time points. At baseline (T_1_), basic participant characteristics were assessed. Active aging (UJACAS-G), physical activity and inactivity, life-space mobility, and health-related quality of life were evaluated by self-administered questionnaires. Physical function was measured by specifically trained assessors (exercise scientists). At follow-up (T_2_), the UJACAS-G was administered a second time. T_2_ was meant to take place between 1 and 3 weeks after T_1_. With the UJACAS-G asking respondents about their experiences over the past 4 weeks, an interval of 1 to 3 weeks was considered short enough to minimize changes in the underlying construct yet long enough to reduce the likelihood of recall. Baseline (T_1_) values were used to explore correlations between UJACAS-G scores and other test results.

The study targeted healthy, community-dwelling older adults (age 65+). A convenience sample of 60 people was recruited for the study; potential participants were approached by study personnel through adult education centers, clubs and service organizations for older adults.

### Inclusion criteria and exclusion criteria

To be eligible for the study, individuals had to fulfil the following inclusion criteria: living in their own home; age 65 or older; ability to walk independently for at least 100 m with or without a walking aid (self-report), and ability to communicate adequately in German. All participants had to provide written informed consent.

A further aim of the present project was to evaluate the reliability of a maximum isometric strength test (‘mid-thigh pull’) [21] in older adults (results to be reported elsewhere). Therefore, history of any of the following health problems—potentially limiting exercise performance or being associated with an increased health risk during high levels of strength exertion—led to exclusion from participation: a musculoskeletal condition limiting exercise ability or performance; ongoing rehabilitation measures after an injury or surgery; back pain (current, in the past 3 months, or chronic); vertebral injury or spinal surgery; osteoporosis or symptoms of osteoporosis (e.g., bone fracture without adequate trauma, marked decrease in body height); a heart problem with medical advice to exercise only under medical supervision; a heart problem under medication; chest pain with exercise; loss of consciousness or falling due to dizziness; untreated arterial hypertension or significantly elevated blood pressure despite antihypertensive medication (>160/>100 mmHg) [21]; current pain at any location; and any other health problem limiting the ability to exercise without medical supervision.

### Measures

#### *UJACAS*—*German version*

The original Finnish version of the UJACAS was translated into German in a collaborative back-and-forth process (involving the developers of the original version) to ensure accuracy and cultural relevance. The process included initial translation (Finnish-German); review by researchers in the fields of gerontology, geriatrics, rehabilitation, sports medicine and exercise science; back translation; comparison with the original Finnish questionnaire and reconciliation; pilot testing; final review and adjustments; and approval [22]. The UJACAS is a 17-item questionnaire covering various activities: memory exercises, computer use, advancing matters in one’s own life, physical exercise, outdoor enjoyment, taking care of one’s appearance, crafting or DIY, home decoration, helping others, maintaining friendships, getting to know new people, financial management, creating interesting days, artistic pursuits, event participation, societal/communal contribution, and doing things according to one’s world view. Participants rate their striving to accomplish each activity, their ability as well as their opportunity to perform each activity, and the recent four-week frequency of doing each activity on a five-point rating scale, from zero (not at all/very low) to four (very much/very high), with specific verbal responses based on each question’s phrasing. The assessment generates subscores (range 0 to 68) for will to act, ability to act, opportunities to act, as well as frequency and volume of doing the activity, plus a total score (sum of the 4 subscores; range 0 to 272). Higher scores indicate a higher level of active aging. Previous research showed that the UJACAS measures a single latent construct of active aging, and has solid psychometric properties, including a good test-retest reliability [10].

#### Physical function

Habitual walking speed was assessed on a 10-meter walkway using a light barrier system (Witty, Microgate Srl, Bolzano, Italy). Walking started 2 m prior to the initial light barrier (to accommodate acceleration) and concluded significantly beyond the finish line (to prevent deceleration within the 10-meter span) [23]. For analysis, the faster of two walking attempts was recorded. Hand grip strength was assessed by dynamometry (Leonardo Mechanograph, Novotec Medical GmbH, Pforzheim, Germany). Participants performed the test standing with full elbow extension, using their dominant hand for three attempts [24]. Grip span was adjusted to fit each participant’s hand size [25]. The highest recorded grip strength was used for analyses. In order to assess postural balance, participants were asked to maintain their feet in the tandem position (heel of one foot directly in front of the other foot) while standing quietly upright (hands on pelvis) on a force platform (Leonardo Mechanograph, Novotec Medical GmbH, Pforzheim, Germany) for 10 s [26]. The total path length of the center of pressure was derived (with lower path length indicating better postural balance); the minimum of three attempts was used. Moreover, participants performed a self-paced six-minute walk test (one attempt) [27]. The test took place on a straight walkway in an indoor corridor with turns every 20 m. The cumulative distance covered during the 6 min was documented.

#### Physical activity and inactivity

Habitual physical activity as well as inactivity (sitting) were assessed using the short version of the International Physical Activity Questionnaire (IPAQ) [28]. This questionnaire assesses the physical activity (days per week and duration on the respective days) of the past 7 days within three domains: vigorous physical activity, moderate physical activity, and walking. A continuous score was calculated (MET level x minutes of activity x days per week) and expressed as MET-min per week. The IPAQ operationalizes inactivity as average sitting time per day. The instrument has demonstrated high reliability and acceptable validity [29, 30].

#### Life-space mobility

Life-space mobility was evaluated by the University of Alabama at Birmingham Study of Aging Life-Space Assessment (LSA) [31]. Participants were instructed to detail the range of their movements over the past four weeks. This range was divided into five spatial levels: (1) rooms in their home outside of the room in which they sleep, (2) the area immediately surrounding their home, (3) their own neighborhood, (4) areas in their town outside their neighborhood, and (5) locations outside their town. Additionally, they were to report how often they travelled to these areas, with options being less than once a week, 1–3 times per week, 4–6 times per week, or daily. They also indicated if they required any form of assistance, such as personal help, use of assistive devices, or no assistance needed. For each spatial level, a subscore was computed by multiplying the given values for the level, frequency of travel, and assistance required. These subscores were then summed to form an overall composite score, which could range from 0 (indicating complete bed confinement) to 120 (denoting daily unassisted travel to out-of-town locations). Higher scores represented greater life-space mobility. The LSA has been consistently recognized for its high reliability, validity, and sensitivity to change [31–33].

#### Health-related quality of life

The Medical Outcomes Study Short Form-36 Health Survey (SF-36™ 4-week recall version) that yields an eight-part profile of functional health and well-being was used to assess health-related quality of life [34]. Two SF-36 composite scores—normalized on population norms, with a mean of 50 and a standard deviation of 10 in the general population—were calculated: the physical component score (PCS) and the mental component score (MCS). Higher scores indicate better quality of life. The instrument has demonstrated good reliability and validity [35, 36].

#### Basic participant characteristics

Sociodemographic factors, including age, sex, living alone, education and financial hardship, were determined through individual self-reporting. The level of education was quantified based on the cumulative years of formal schooling and vocational training. The assessment of financial hardship involved querying whether the individual faced financial challenges that complicated their everyday life (participation) in the preceding four weeks. Responses ranged from ‘no impact’, ‘complicated life somewhat’ to ‘complicated life massively’ [37]. A trained assessor measured weight and height, from which body mass index was computed. The presence of health problems (heart disease, high blood pressure, lung disease, diabetes, ulcer or stomach disease, kidney disease, liver disease, anemia or other blood disease, cancer, depression, osteoarthritis/degenerative arthritis, back pain, rheumatoid arthritis) was assessed by the Self-Administered Comorbidity Questionnaire (SCQ) (“Do you have the problem?” yes vs no) [38]. In order to assess mobility limitation, participants were queried regarding their use of a walking aid as well as their ability to walk 2 km and ascend 1 flight of stairs [39]. Available responses included ‘Yes, without difficulty’; ‘Yes, but with some difficulty’; ‘Yes, but with a great deal of difficulty’; ‘Yes, but not without help’; and ‘Not even with help’. The frequency of falls (number of falls with the previous 12 months) was also assessed by self-report [40].

### Sample size

Sample size calculation was based on Bland-Altman analyses for agreement between assessments performed at T_1_ and T_2_ (test-retest). Independent of the outcome parameter used, a sample size of 47 results in an accuracy of ±0.5*s for the estimation of limits of agreement, where *s* is the standard deviation of the differences between measurements performed at T_1_ and T_2_ [41, 42]. Accounting for a drop-out of 20% between T_1_ and T_2_, the target sample size was 60.

### Statistical analyses

Participant characteristics, UJACAS-G scores (at T_1_) as well as parameters of physical function, physical activity, life-space mobility, and health-related quality of life were analyzed descriptively (numbers, percentages, means, standard deviations, medians, and interquartile ranges as appropriate). To determine test-retest reliability, UJACAS-G scores of T_1_ and T_2_ were assessed for agreement by performing Bland-Altman analyses and by calculating ICCs (type A,1) [41, 43]. To explore concurrent validity, correlations (Spearman’s rho) between UJACAS-G scores and parameters of physical function, physical activity, life-space mobility, and health-related quality of life were calculated (strength of relationship: less than 0.3—poor; 0.3 to 0.5—fair; 0.6 up to 0.8 moderately strong; at least 0.8—very strong [44]). IBM SPSS Statistics 28 (IBM Inc., Armonk, NY, USA) was used for statistical analyses; the level of significance was set at α = 0.05.

## Results

### Translation process

The translation process led to a number of adaptations between first translation and final German version (Table 1). No major cross-cultural differences were identified with regard to the activities addressed in the questionnaire. The final German version is provided as supplementary online material.

**Table 1:** Adaptations between first and final German version of the University of Jyvaskyla Active Aging Scale (UJACAS)

Item	Finnish version (original)	English version	First German version	Final German version
1–17	Toimintakyky	Capacity	Leistungsfähigkeit	Funktionsfähigkeit
1–8	Toiminnan useus	Frequency of doing	Durchführungshäufigkeit	Häufigkeit des Handelns
1	Käsityöt, nikkarointi tai muiden kädentaitojen harrastaminen	Crafting, DIY or other pastimes requiring manual skills	Handarbeiten ausführen, Basteln oder anderen Freizeitbeschäftigungen nachgehen, die Handfertigkeit erfordern	Handarbeiten ausführen, Heimwerken oder anderen Freizeitbeschäftigungen nachgehen, die Handfertigkeit erfordern
5	Kuntoilu	To practice keeping physically fit	Trainieren, um mich körperlich fit zu halten	Körperlich aktiv sein, um mich fit zu halten
12	Vastuun ottaminen yhteiskunnallisten tai yhteisöllisten asioiden edistämiseksi	To take responsibility for promoting societal or public matters	Verantwortung für die Förderung gesellschaftlicher oder öffentlicher Angelegenheiten übernehmen	Verantwortung für die Förderung gesellschaftlicher Angelegenheiten oder Gemeindeangelegenheiten übernehmen
16	Taloudellisen tilanteen tasapainosta huolehtiminen	To ensure that my financial affairs are in order	Sicherstellen, dass meine finanziellen Angelegenheiten in Ordnung sind	Mich darum kümmern, dass meine finanziellen Angelegenheiten in Ordnung sind
17	Asioiden tekeminen vakaumuksen tai maailmankatsomuksen eteen	To take action to further matters according to my faith or worldview	Massnahmen ergreifen, um Angelegenheiten meines Glaubens und meiner Weltanschauung voranzutreiben	Mit Dingen beschäftigen, die meinen Glauben oder meine Weltanschauung betreffen

### Participants

Sixty older adults (age range 65 to 93, mean 72.3, SD 5.9 years; 50% women) participated in the study. No participant dropped out between T_1_ and T_2_. Time between T_1_ and T_2_ ranged between 7 and 21 (mean 10.8; median 10; SD 3.7) days. On average, participants had a high socio-economic status. The prevalence of chronic health conditions, mobility limitations, and falls was low (Table 2). Descriptive statistics of measures of active aging, physical function, physical activity, life-space mobility, and health-related quality of life at T_1_ are shown in Table 3.

**Table 2:** Basic participant characteristics (*N* = 60)

Characteristic	*N*	*n* (%)	Mean (SD)	Median (IQR)
**Sociodemographics**				
Age	60		72.3 (5.9)	71.0 (67.25; 76.0)
Female	60	30 (50.0)		
Living alone	60	13 (21.7)		
Years of education	60		14.6 (3.1)	13.5 (13.0; 17.0)
Financial hardship	60			
No difficulties		59 (98.3)		
Some difficulties		1 (1.7)		
**Health-related parameters**				
Body Mass Index [kg/m^2^]	60		25.0 (3.6)	24.6 (22.1; 27.4)
Heart disease (yes)	60	3 (5.0)		
High blood pressure (yes)	60	10 (16.7)		
Lung disease (yes)	60	5 (8.3)		
Diabetes (yes)	60	1 (1.7)		
Ulcer or stomach disease (yes)	60	1 (1.7)		
Kidney disease (yes)	60	0		
Liver disease (yes)	60	0		
Anemia or other blood disease (yes)	60	4 (6.7)		
Cancer (yes)	60	2 (3.3)		
Depression (yes)	60	3 (5.0)		
Osteoarthritis/degenerative arthritis (yes)	60	13 (21.7)		
Back pain (yes)	60	11 (18.3)		
Rheumatoid arthritis (yes)	60	2 (3.3)		
Difficulties walking 2 km	60			
No difficulties		59 (98.3)		
Some difficulties		1 (1.7)		
Difficulties climbing 1 flight of stairs	60			
No difficulties		59 (98.3)		
Some difficulties		1 (1.7)		
Number of falls in past 12 months	60			
0		44 (73.3)		
1		14 (23.3)		
≥2		2 (3.3)		
Regular use of walking aid outdoors	60	0 (100)		

*SD* standard deviation, *IQR* interquartile range

**Table 3:** Descriptive statistics of active aging, physical function, physical activity, life-space mobility, and health-related quality of life at T_1_

Characteristic	*N*	Mean (SD)	Median (IQR)
**Active aging**			
UJACAS-G total score (0–272)	60	208.5 (22.7)	206.5 (198.0; 222.75)
Goals subscore (0–68)	60	44.6 (9.2)	46.0 (38.0; 51.0)
Ability subscore (0–68)	60	65.3 (4.0)	67.0 (64.0; 68.0)
Opportunity subscore (0–68)	60	55.8 (6.7)	55.5 (51.25; 60.0)
Activity subscore (0–68)	60	42.8 (9.3)	43.0 (35.0; 49.0)
**Physical function**			
Ten meter habitual walking speed (m/s)	58	1.48 (0.17)	1.47 (1.34; 1.61)
Handgrip strength (N)	59	328.0 (105.6)	307.1 (238.4; 431.6)
Postural balance (cm)^a^	59	369.2 (144.4)	354 (248.3; 432)
Six-minute walk distance (m)	59	596.6 (77.0)	592 (536; 650)
**Habitual physical activity**			
IPAQ-Short activity (active MET-minutes/week)	59	5253.1 (3514.4)	4479 (2748; 7092)
IPAQ-Short inactivity (minutes of sitting/day)	60	301.5 (129.6)	300 (187.5; 360)
**Life-space mobility**			
UAB-LSA composite score (0–120)	60	96.6 (16.7)	100 (86.5; 110)
**Health-related quality of life**			
SF-36 Physical component score	60	52.0 (3.4)	52.5 (49.8; 54.4)
SF-36 Mental component score	60	54.5 (5.6)	55.9 (52.4; 58.1)

*UJACAS-G* University of Jyvaskyla Active Aging Scale-German Version, *IPAQ* International Physical Activity Questionnaire, *SF-36* Medical Outcomes Study 36-Item Short Form Health Survey, *SD* standard deviation, *IQR* interquartile range

^a^Total path length of center of pressure in tandem stance

### Test-retest reliability

**Fig. 1:**
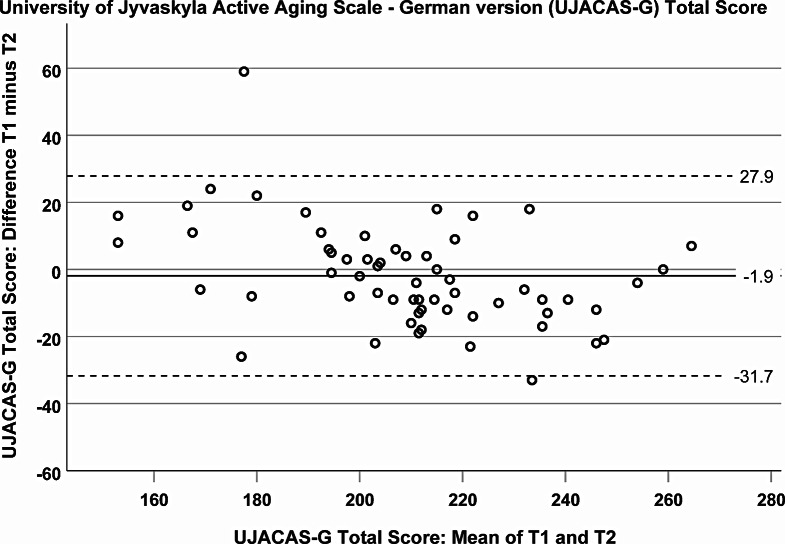
Bland-Altman plots illustrating the agreement between T_1_ and T_2_ of the German version of the University of Jyvaskyla Active Aging Scale (UJACAS-G) total score. The continuous horizontal line shows the mean difference between measurements at the two time points (T_1_ minus T_2_); the dashed lines show the limits of agreement, defined as the mean difference plus and minus 1.96 times the standard deviation of the differences

**Fig. 2:**
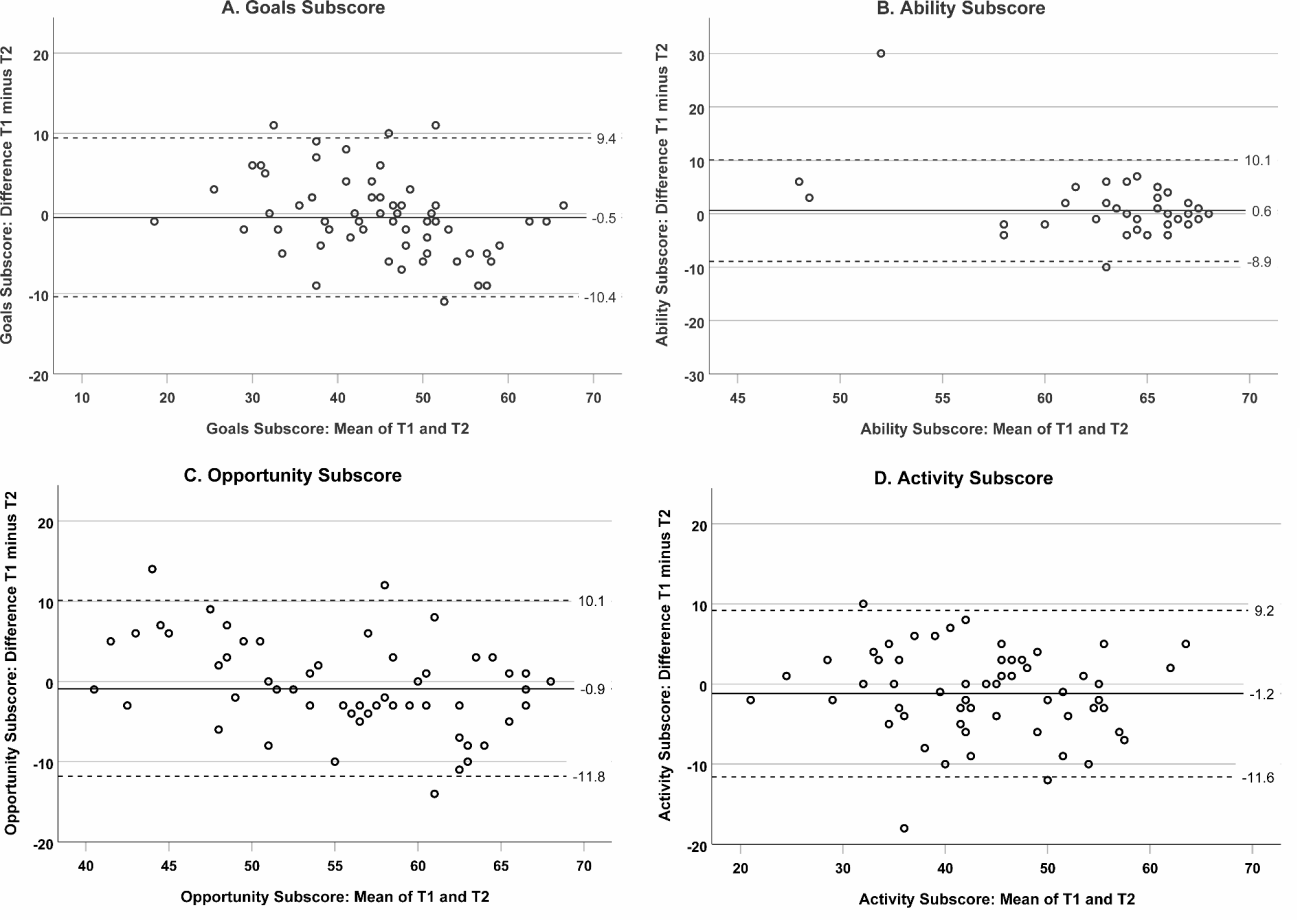
**A–D** Bland-Altman plots illustrating the agreement between T_1_ and T_2_ of the German version of the University of Jyvaskyla Active Aging Scale (UJACAS-G) subscores goals (**A**), ability (**B**), opportunity (**C**), and activity (**D**)

Bland-Altman analyses were employed to evaluate the agreement between the measurement at T_1_ and the measurement at T_2_. Analyses showed that the mean difference between the two sets of measurements (representing the systematic bias between the measurements) was close to zero, suggesting good agreement; this refers to the total score (Fig. 1) as well as to the four subscores (Fig. 2A–D). Furthermore, limits of agreement (defined as the mean difference ± 1.96 standard deviations) were narrow for the total score (upper limit 27.9; lower limit −31.7) as well as for the subscores. It should be noted that in the ability subscore, a relevant number of participants (*n* = 17; 28.3%) achieved the maximum score in both measurements, suggesting a ‘ceiling effect’ in our sample; i.e., measurements were unable to distinguish performance differences at the higher end of the scale. For this subscore, the mean difference and the limits of agreement may be underestimated due to the observed ‘ceiling effect’, and thus, their interpretation requires careful consideration of this bias. No ‘ceiling effect’ was observed for the total score. Notably, the scatter plots for the total score, as well as for the goals, opportunity, and activity subscores, demonstrated random distributions of differences across the range of measurements, with no proportional bias observed, indicating that discrepancies did not vary systematically across the magnitude of measurements. Besides clustering at the upper limit, the distribution scatterplot for the ability subscore exhibited a ‘trumpet’ shape, with an increasing spread of differences towards the lower end of the scale. The scatterplot additionally highlighted a significant outlier characterized by an unusually large disparity between the T_2_ and T_1_ ability subscore (Fig. 2B). Upon reviewing the individual records, no clear reason (such as an acute health issue) was found to account for this marked inconsistency in response behavior.

ICCs for UJACAS-G total score, goals subscore and activity subscore ranged between 0.829 and 0.876 (Table 4) and were thereby interpretable as ‘good reliability’ according to Koo and Li [45]. ICCs for ability subscore and opportunity subscore were 0.530 and 0.747, respectively and thereby interpretable as ‘moderate reliability’; again with the ability subscore to be interpreted with caution due to its skewed distribution. In a sensitivity analysis excluding the outlier in the ability subscore, the ICC (*n* = 59) for the ability subscore was 0.769 (95% confidence interval 0.639 to 0.856; *p* < 0.001), and the ICC for the total score was 0.866 (95% confidence interval 0.784 to 0.918; *p* < 0.001).

**Table 4:** Intraclass correlations (T1 vs. T2) as measures of test-retest reliability of the University of Jyvaskyla Active Aging Scale (UJACAS-G) total score and its subscores

Measure	*N*	ICC	95% CI		*p*
			Lower	Upper	
UJACAS-G total score	60	0.829	0.730	0.894	<0.001
Goals subscore	60	0.876	0.800	0.924	<0.001
Ability subscore	60	0.530	0.321	0.689	<0.001
Opportunity subscore	60	0.747	0.611	0.840	<0.001
Activity subscore	60	0.840	0.746	0.902	<0.001

*UJACAS-G* University of Jyvaskyla Active Aging Scale-German Version, *ICC* intraclass correlation coefficient, *CI* confidence interval

### Concurrent validity

Correlations of the UJACAS-G total score and its subscores with parameters of physical function, physical activity, life-space mobility and health-related quality of life are shown in Table 5. Only poor to fair correlations were identified, as indicated by the correlation coefficient values. The UJACAS-G total score correlated fairly with habitual walking speed (Spearman’s rho 0.345; *p* = 0.008), physical activity (Spearman’s rho 0.279; *p* = 0.033) and mental health (Spearman’s rho 0.329; *p* = 0.010).

**Table 5:** Correlations between University of Jyvaskyla Active Aging Scale (UJACAS-G) scores and measures of physical function, physical activity, life space mobility, and health-related quality of life

Measure	*N*	UJACAS-G total score		Goals subscore		Ability subscore		Opportunity subscore		Activity subscore	
		Spearman’s rho	*p*	Spearman’s rho	*p*	Spearman’s rho	*p*	Spearman’s rho	*p*	Spearman’s rho	*p*
**Physical function**											
Ten meter habitual walking speed (m/s)	58	0.345	**0.008**	0.312	**0.017**	0.358	**0.006**	0.382	**0.003**	0.192	0.148
Handgrip strength (N)	59	0.235	0.073	0.187	0.155	0.255	0.051	0.292	**0.025**	0.079	0.554
Postural balance (cm)^a^	59	−0.213	0.105	−0.253	0.053	−0.343	**0.008**	−0.135	0.307	−0.181	0.171
Six minute walk distance (m)	59	0.151	0.255	0.131	0.323	0.214	0.103	0.293	**0.024**	0.059	0.659
**Habitual physical activity and inactivity**											
IPAQ-Short activity (active MET-minutes/week)	59	0.279	**0.033**	0.262	**0.045**	−0.113	0.395	0.057	0.666	0.398	**0.002**
IPAQ-Short inactivity (minutes of sitting/day)	60	−0.119	0.363	−0.097	0.462	0.189	0.148	−0.080	0.543	−0.175	0.180
**Life-space mobility**											
UAB-LSA composite score (0–120)	60	0.116	0.376	0.121	0.355	0.315	**0.014**	0.100	0.445	0.091	0.488
**Health-related quality of life**											
SF-36 Physical health summary scale	60	0.181	0.165	0.117	0.372	0.168	0.200	0.220	0.092	0.081	0.538
SF-36 Mental health summary scale	60	0.329	**0.010**	0.246	0.058	0.406	**0.001**	0.294	**0.022**	0.189	0.148

^a^Total path length of center of pressure in semitandem stance; higher path length indicating lower postural balance

*p*-values ≤ 0.05 are bolded

## Discussion

The German version of the UJACAS demonstrated good reliability in a healthy older adult population, as evidenced by strong agreement in Bland-Altman analyses as well as moderate to high ICCs for measurements conducted approximately 11 days apart. This applies to both the total score and the individual subscores. Furthermore, exploratory analysis of correlations with health-related parameters revealed only weak associations.

### Test-retest reliability

We employed Bland-Altman analyses to assess the agreement between repeated measurements of the UJACAS-G. This method provides a graphical representation of the differences between measurements, allowing us to identify any systematic bias and the limits of agreement. Our Bland-Altman plots indicated that the majority of the differences between repeated measurements fell within the acceptable range, demonstrating good agreement and supporting the test-retest-reliability of the UJACAS-G. While the previous—Finnish [10], Turkish [13], and Swedish [12] studies did not perform Bland-Altman analyses, they reported test-retest reliability through ICCs. Within the developmental process at the University of Jyväskylä, the original UJACAS was tested for test-retest reliability—with tests approximately two weeks apart—in a convenience sample of 67 older adults, aged 65 to 86 [10]. Similar to the present study, authors observed a clustering of values at the upper end of the scale for the ability dimension (with 17% of the sample receiving the maximum score [10]); however, in line with present findings, there was no apparent clustering toward minimum or maximum values within the total score or the other subscores. For the original UJACAS [10], ICC was 0.915 for the total score and ranged between 0.885 and 0.928 for the subscores (all *p*-values < 0.001), indicating good to excellent reliability. Demir Erbil and Hazer [13] adapted the UJACAS to Turkish and applied it to 25 older adults twice with a 3-week interval. Correlations between test and retest (Pearson’s *r*) were *r* = 0.91 for the total score, and ranged between *r* = 0.90 and *r* = 0.92 for the subscores. Nordeström et al. [12] adapted the UJACAS to Swedish and investigated test-retest reliability in a convenience sample of 63 older adults, aged 61 to 92. They found an ICC of 0.88 for the total score; ICCs for the subscores ranged between 0.71 (opportunity) and 0.90 (activity). In conclusion, our results are in line with previous test-retest studies, consistently showing a high test-retest reliability of the original UJACAS and its adaptations.

### Concurrent validity

Acknowledging the lack of a ‘gold standard’ for assessing active aging, the development of the original UJACAS involved an occupational therapist conducting interviews with 45 older individuals in order to establish the scale’s validity [10]. Interviews focused on the participants’ daily activities, with an emphasis on identifying activities they found meaningful and any they wished to engage in but were unable to, or chose not to. Additionally, participants were encouraged to express their functional capabilities in their own terms. Following these discussions, the therapist assigned each participant a score from 0, indicating no activity, to 10, representing the highest degree of activity and involvement in meaningful tasks. The pilot study showed a moderate correlation (Pearson’s *r* 0.658; *p* < 0.001) between the occupational therapist’s assessment and the UJACAS total score; correlation coefficients for the subscores ranged between *r* = 0.476 (goals) and *r* = 0.681 (ability) [10]. In a larger sample of *N* = 155 older adults, authors identified mostly fair positive correlations between UJACAS scores and goals in life, autonomy, self-rated health, quality of life, and life-space mobility (Pearson’s *r* for total score and subscores ranging between 0.268 and 0.612; all *p*-values < 0.001). Negative correlations were found between UJACAS (total score and subscores) and perceiving the own poor health as a barrier for active aging as well as perceiving poor opportunities for active aging (Person’s *r* ranging between −0.162 and −0.704; all *p*-values < 0.05) [10]. For the Swedish version of the UJACAS, Nordeström et al. [12] found that higher UJACAS scores correlated with higher self-rated health (*r* = 0.41; *p* < 0.01) and with higher life-space mobility (*r* = 0.24; *p* < 0.01). Given the previously noted lack of a ‘gold standard’, our approach involved exploring the correlations between UJACAS-G scores and a wide array of health-related factors. Our findings revealed predominantly weak associations, with only a select few demonstrating statistical significance at the level of *p* ≤ 0.05. The weak correlations observed may result from our sample of healthy older adults, who had consistently high levels of physical function and quality of life, reducing the variability needed for stronger associations.

### Strengths and limitations

This is the first study to make the UJACAS available for research on active aging in German-speaking populations. The scale was originally developed for older people, regardless of their health and functional status [10]. While previous validity and reliability studies included diverse samples without limiting participants to those in good health [10, 12, 13], our sample consisted only of healthy older adults with a high physical function. In consequence, (a) our results are not directly transferrable to chronically ill, inactive or physically impaired populations, and (b) due to the lower variance in a more homogeneous sample, reliability and validity may appear lower than they actually are. A further limitation refers to the time interval between T_1_ and T_2_; for practical reasons (i.e., the availability of participants) we allowed an interval of 1 to 3 weeks between the two visits at the study center. While shorter intervals increase the risk of recall bias, longer intervals increase the risk that any genuine changes in the respondents’ condition or attitudes rather than measurement error influence the responses. Another limitation of our study is the small sample size, which is sufficient for identifying major issues but limits the ability to conduct advanced psychometric analyses necessary for comparing the original Finnish version with the translated German version. Future research with larger sample sizes is needed to perform these advanced analyses.

## Conclusions

The UJACAS-G is a reliable tool that can be used in future studies in German-speaking healthy older populations; its psychometric properties in chronically diseased and mobility-limited older populations will have to be explored. A potential ‘ceiling effect’ regarding the ability subscore should be considered when applying the UJACAS-G to well-functioning populations. Exploratory analysis of correlations with health-related parameters revealed only weak associations.

## Electronic supplementary material

Below is the link to the electronic supplementary material.

Supplementary Material 1

## Data Availability

The datasets are available from the corresponding author on reasonable request. The German version of the UJACAS is available as online supplementary material to this publication. The English version is available at: www.gerec.fi/en/agnes/ujacas.

## Abbreviations

ICC: Intraclass correlation coefficient
ICF: International Classification of Functioning, Disability, and Health
IPAQ: International Physical Activity Questionnaire
LSA: University of Alabama at Birmingham Study of Aging Life-Space Assessment
MCS: Mental component score
MET: Metabolic equivalent
PCS: Physical component score
SCQ: Self-Administered Comorbidity Questionnaire
SD: Standard deviation
SF-36: Medical Outcomes Study Short Form-36 Health Survey
UJACAS: University of Jyvaskyla Active Aging Scale
UJACAS-G: German version of the University of Jyvaskyla Active Aging Scale
WHO: World Health Organization
