# Supplementary material for: Reliability and validity of the German version of the University of Jyvaskyla Active Aging Scale (UJACAS-G)
Source: J Patient Rep Outcomes. 2024 Sep 10;8:104. doi: 10.1186/s41687-024-00786-w (PMC11387280; doi:10.1186/s41687-024-00786-w)
Supplement: Supplementary file 1 — Supplementary Material 1 [file 41687_2024_786_MOESM1_ESM.pdf]

JYVÄSKYLÄN YLIOPISTO
UNIVERSITY OF JYVÄSKYLÄ

GEREC
GERONTOLOGY RESEARCH CENTER

University of Basel

University of Jyvaskyla Active Aging Scale – German Version (UJACAS-G)

Aktiivinen vanhuus Agnes Tutkimus

Auf den folgenden Seiten sehen Sie einen Fragebogen. Ziel dieses Fragebogens ist es herauszufinden, ob Sie an bestimmten alltäglichen Aktivitäten interessiert sind und welche Möglichkeiten Sie haben, diesen Aktivitäten nachzugehen. Der Fragebogen fragt nach 17 verschiedenen Aktivitäten aus vier verschiedenen Perspektiven. Denken Sie bei der Beantwortung der Fragen an Ihr Leben **in den letzten vier Wochen**.

Die vier Perspektiven lauten wie folgt:
WILLE ZU HANDELN: Bewerten Sie, wie sehr Sie die fraglichen Aktivitäten in den letzten vier Wochen durchführen wollten.
HANDLUNGSFÄHIGKEIT: Bewerten Sie, **unter Berücksichtigung Ihres Gesundheitszustandes und Ihrer Funktionsfähigkeit**, wie gut Sie in der Lage waren oder gewesen wären, die betreffenden Aktivitäten durchzuführen.
MÖGLICHKEIT ZU HANDELN: Wenn Sie über Ihr **Leben im Allgemeinen** nachdenken, bewerten Sie, wie Sie in den letzten vier Wochen Ihre Möglichkeiten zur Durchführung der betreffenden Aktivitäten erlebt haben.
HÄUFIGKEIT / AUSMASS DES HANDELNS: Geben Sie an, wie oft oder wie viel Sie die betreffenden Aktivitäten in den letzten vier Wochen ausgeführt haben.

Kreuzen Sie für jede Frage (1-17) in jeder der vier Spalten (**WILLE ZU HANDELN**, **HANDLUNGSFÄHIGKEIT**, **MÖGLICHKEIT ZU HANDELN**, **HÄUFIGKEIT / AUSMASS DES HANDELN**) eine Alternative an.

Beantworten Sie jede Aussage (1-17)

	**WILLE ZU HANDELN**<br>Wie sehr wollten Sie **in den letzten vier Wochen** die folgenden Dinge tun?	**HANDLUNGSFÄHIGKEIT**<br>**Unter Berücksichtigung Ihres Gesundheitszustandes und Ihrer Funktionsfähigkeit**, waren Sie **in den letzten vier Wochen** in der Lage oder wären Sie in der Lage gewesen, die folgenden Dinge zu tun?	**MÖGLICHKEIT ZU HANDELN**<br>Wenn Sie über **Ihr Leben im Allgemeinen** nachdenken, wie haben Sie **in den letzten vier Wochen** Ihre Möglichkeiten zur Durchführung der folgenden Dinge erlebt?	**HÄUFIGKEIT DES HANDELNS**<br>Wie häufig haben Sie die folgenden Dinge in den **letzten vier Wochen** getan?
**1**	Ich wollte Handarbeiten ausführen, heimwerken oder anderen Freizeitbeschäftigungen nachgehen, die Handfertigkeit erfordern<br>○ 1. Sehr stark<br>○ 2. Ziemlich stark<br>○ 3. Zu einem gewissen Grad<br>○ 4. Nur ein bisschen<br>○ 5. Überhaupt nicht	Ich war in der Lage oder wäre in der Lage gewesen, Handarbeiten auszuführen, zu heimwerken oder anderen Freizeitbeschäftigungen nachzugehen, die Handfertigkeit erfordern<br>○ 1. Ja, ohne Schwierigkeiten<br>○ 2. Ja, aber mit gewissen Schwierigkeiten<br>○ 3. Ja, aber mit grossen Schwierigkeiten<br>○ 4. Nicht ohne fremde Hilfe<br>○ 5. Nicht einmal mit Hilfe	Meine Möglichkeiten Handarbeiten auszuführen, zu heimwerken oder anderen Freizeitbeschäftigungen nachzugehen, die Handfertigkeit erfordern waren<br>○ 1. Sehr gut<br>○ 2. Ziemlich gut<br>○ 3. Mittelmässig<br>○ 4. Eingeschränkt<br>○ 5. Es war nicht möglich	Ich habe Handarbeiten ausgeführt, heimgewerkt oder bin anderen Freizeitbeschäftigungen nachgegangen, die Handfertigkeit erfordern<br>○ 1. Täglich oder fast täglich<br>○ 2. 2-4 mal pro Woche<br>○ 3. Etwa einmal pro Woche<br>○ 4. Weniger als einmal pro Woche<br>○ 5. Überhaupt nicht
**2**	Ich wollte zeichnen, ein Musikinstrument spielen, singen, schreiben oder eine andere künstlerische Beschäftigung ausüben<br>○ 1. Sehr stark<br>○ 2. Ziemlich stark<br>○ 3. Zu einem gewissen Grad<br>○ 4. Nur ein bisschen<br>○ 5. Überhaupt nicht	Ich war in der Lage oder wäre in der Lage gewesen zu zeichnen, ein Musikinstrument zu spielen, zu singen, zu schreiben oder eine andere künstlerische Beschäftigung auszuüben<br>○ 1. Ja, ohne Schwierigkeiten<br>○ 2. Ja, aber mit gewissen Schwierigkeiten<br>○ 3. Ja, aber mit grossen Schwierigkeiten<br>○ 4. Nicht ohne fremde Hilfe<br>○ 5. Nicht einmal mit Hilfe	Meine Möglichkeiten zu zeichnen, zu singen oder ein Musikinstrument zu spielen, zu schreiben oder eine andere künstlerische Beschäftigung auszuüben waren<br>○ 1. Sehr gut<br>○ 2. Ziemlich gut<br>○ 3. Mittelmässig<br>○ 4. Eingeschränkt<br>○ 5. Es war nicht möglich	Ich habe gezeichnet, ein Musikinstrument gespielt, gesungen, geschrieben oder habe eine andere künstlerische Beschäftigung ausgeübt<br>○ 1. Täglich oder fast täglich<br>○ 2. 2-4 mal pro Woche<br>○ 3. Etwa einmal pro Woche<br>○ 4. Weniger als einmal pro Woche<br>○ 5. Überhaupt nicht
**3**	Ich wollte an verschiedenen Veranstaltungen oder Aktivitäten teilnehmen, die mit Bildung, mit Vereinen oder Verbänden zu tun haben<br>○ 1. Sehr stark<br>○ 2. Ziemlich stark<br>○ 3. Zu einem gewissen Grad<br>○ 4. Nur ein bisschen<br>○ 5. Überhaupt nicht	Ich war in der Lage oder wäre in der Lage gewesen an verschiedenen Veranstaltungen oder Aktivitäten teilzunehmen, die mit Bildung, mit Vereinen oder Verbänden zu tun haben<br>○ 1. Ja, ohne Schwierigkeiten<br>○ 2. Ja, aber mit gewissen Schwierigkeiten<br>○ 3. Ja, aber mit grossen Schwierigkeiten<br>○ 4. Nicht ohne fremde Hilfe<br>○ 5. Nicht einmal mit Hilfe	Meine Möglichkeiten an verschiedenen Veranstaltungen oder Aktivitäten teilnehmen, die mit Bildung, mit Vereinen oder Verbänden zu tun haben<br>○ 1. Sehr gut<br>○ 2. Ziemlich gut<br>○ 3. Mittelmässig<br>○ 4. Eingeschränkt<br>○ 5. Es war nicht möglich	Ich habe an verschiedenen Veranstaltungen oder Aktivitäten teilgenommen, die mit Bildung, mit Vereinen oder Verbänden zu tun haben<br>○ 1. Täglich oder fast täglich<br>○ 2. 2-4 mal pro Woche<br>○ 3. Etwa einmal pro Woche<br>○ 4. Weniger als einmal pro Woche<br>○ 5. Überhaupt nicht
**4**	Ich wollte nach draussen gehen und die Natur geniessen<br>○ 1. Sehr stark<br>○ 2. Ziemlich stark<br>○ 3. Zu einem gewissen Grad<br>○ 4. Nur ein bisschen<br>○ 5. Überhaupt nicht	Ich war in der Lage oder wäre in der Lage gewesen nach draussen zu gehen und die Natur zu geniessen<br>○ 1. Ja, ohne Schwierigkeiten<br>○ 2. Ja, aber mit gewissen Schwierigkeiten<br>○ 3. Ja, aber mit grossen Schwierigkeiten<br>○ 4. Nicht ohne fremde Hilfe<br>○ 5. Nicht einmal mit Hilfe	Meine Möglichkeiten nach draussen zu gehen und die Natur zu geniessen waren<br>○ 1. Sehr gut<br>○ 2. Ziemlich gut<br>○ 3. Mittelmässig<br>○ 4. Eingeschränkt<br>○ 5. Es war nicht möglich	Ich bin nach draussen gegangen und habe die Natur genossen<br>○ 1. Täglich oder fast täglich<br>○ 2. 2-4 mal pro Woche<br>○ 3. Etwa einmal pro Woche<br>○ 4. Weniger als einmal pro Woche<br>○ 5. Überhaupt nicht

Beantworten Sie jede Aussage (1-17)

	**WILLE ZU HANDELN**<br>Wie sehr wollten Sie **in den letzten vier Wochen** die folgenden Dinge tun?	**HANDLUNGSFÄHIGKEIT**<br>Haben oder hätten Sie, **unter Berücksichtigung Ihres Gesundheitszustands und Ihrer Funktionsfähigkeit**, **in den letzten vier Wochen Folgendes getan**?	**MÖGLICHKEIT ZU HANDELN**<br>Wenn Sie über **Ihr Leben im Allgemeinen** nachdenken, wie haben Sie **in den letzten vier Wochen** Ihre Möglichkeiten zur Durchführung der folgenden Dinge erlebt?	**HÄUFIGKEIT DES HANDELNS**<br>Geben Sie an, wie häufig Sie die betreffenden Aktivitäten in den **letzten vier Wochen** ausgeführt haben.
**5**	Ich wollte körperlich aktiv sein, um mich fit zu halten<br>○ 1. Sehr stark<br>○ 2. Ziemlich stark<br>○ 3. Zu einem gewissen Grad<br>○ 4. Nur ein bisschen<br>○ 5. Überhaupt nicht	Ich war oder wäre in der Lage gewesen körperlich aktiv sein, um mich fit zu halten<br>○ 1. Ja, ohne Schwierigkeiten<br>○ 2. Ja, aber mit gewissen Schwierigkeiten<br>○ 3. Ja, aber mit grossen Schwierigkeiten<br>○ 4. Nicht ohne fremde Hilfe<br>○ 5. Nicht einmal mit Hilfe	Meine Möglichkeiten körperlich aktiv sein, um mich fit zu halten waren<br>○ 1. Sehr gut<br>○ 2. Ziemlich gut<br>○ 3. Mittelmässig<br>○ 4. Eingeschränkt<br>○ 5. Es war nicht möglich	Ich war körperlich aktiv, um mich fit zu halten<br>○ 1. Täglich oder fast täglich<br>○ 2. 2-4 mal pro Woche<br>○ 3. Etwa einmal pro Woche<br>○ 4. Weniger als einmal pro Woche<br>○ 5. Überhaupt nicht
**6**	Ich wollte Übungen machen, um meinen Verstand und mein Gedächtnis zu trainieren<br>○ 1. Sehr stark<br>○ 2. Ziemlich stark<br>○ 3. Zu einem gewissen Grad<br>○ 4. Nur ein bisschen<br>○ 5. Überhaupt nicht	Ich war oder wäre in der Lage gewesen Übungen zu machen, um meinen Verstand und mein Gedächtnis zu trainieren<br>○ 1. Ja, ohne Schwierigkeiten<br>○ 2. Ja, aber mit gewissen Schwierigkeiten<br>○ 3. Ja, aber mit grossen Schwierigkeiten<br>○ 4. Nicht ohne fremde Hilfe<br>○ 5. Nicht einmal mit Hilfe	Meine Möglichkeiten Übungen zu machen, um meinen Verstand und mein Gedächtnis zu trainieren waren<br>○ 1. Sehr gut<br>○ 2. Ziemlich gut<br>○ 3. Mittelmässig<br>○ 4. Eingeschränkt<br>○ 5. Es war nicht möglich	Ich habe Übungen gemacht, um meinen Verstand und mein Gedächtnis zu trainieren<br>○ 1. Täglich oder fast täglich<br>○ 2. 2-4 mal pro Woche<br>○ 3. Etwa einmal pro Woche<br>○ 4. Weniger als einmal pro Woche<br>○ 5. Überhaupt nicht
**7**	Ich wollte einen Computer oder ein Tablet verwenden<br>○ 1. Sehr stark<br>○ 2. Ziemlich stark<br>○ 3. Zu einem gewissen Grad<br>○ 4. Nur ein bisschen<br>○ 5. Überhaupt nicht	Ich war oder wäre in der Lage gewesen einen Computer oder ein Tablet zu verwenden<br>○ 1. Ja, ohne Schwierigkeiten<br>○ 2. Ja, aber mit gewissen Schwierigkeiten<br>○ 3. Ja, aber mit grossen Schwierigkeiten<br>○ 4. Nicht ohne fremde Hilfe<br>○ 5. Nicht einmal mit Hilfe	Meine Möglichkeiten einen Computer oder ein Tablet zu verwenden waren<br>○ 1. Sehr gut<br>○ 2. Ziemlich gut<br>○ 3. Mittelmässig<br>○ 4. Eingeschränkt<br>○ 5. Es war nicht möglich	Ich habe einen Computer oder ein Tablet verwendet<br>○ 1. Täglich oder fast täglich<br>○ 2. 2-4 mal pro Woche<br>○ 3. Etwa einmal pro Woche<br>○ 4. Weniger als einmal pro Woche<br>○ 5. Überhaupt nicht
**8**	Ich wollte Menschen die mir nahestehen oder anderen Menschen helfen oder sie unterstützen<br>○ 1. Sehr stark<br>○ 2. Ziemlich stark<br>○ 3. Zu einem gewissen Grad<br>○ 4. Nur ein bisschen<br>○ 5. Überhaupt nicht	Ich war oder wäre in der Lage gewesen Menschen die mir nahestehen oder anderen Menschen zu helfen oder sie zu unterstützen<br>○ 1. Ja, ohne Schwierigkeiten<br>○ 2. Ja, aber mit gewissen Schwierigkeiten<br>○ 3. Ja, aber mit grossen Schwierigkeiten<br>○ 4. Nicht ohne fremde Hilfe<br>○ 5. Nicht einmal mit Hilfe	Meine Möglichkeiten Menschen die mir nahestehen oder anderen Menschen zu helfen oder sie zu unterstützen waren<br>○ 1. Sehr gut<br>○ 2. Ziemlich gut<br>○ 3. Mittelmässig<br>○ 4. Eingeschränkt<br>○ 5. Es war nicht möglich	Ich habe Menschen die mir nahestehen oder anderen Menschen geholfen oder sie unterstützt<br>○ 1. Täglich oder fast täglich<br>○ 2. 2-4 mal pro Woche<br>○ 3. Etwa einmal pro Woche<br>○ 4. Weniger als einmal pro Woche<br>○ 5. Überhaupt nicht

In den folgenden Fragen interessiert uns, wie sehr und nicht wie oft Sie die folgenden Dinge getan haben

Beantworten Sie jede Aussage (1-17)

	**WILLE ZU HANDELN**<br>Wie sehr wollten Sie **in den letzten vier Wochen** die folgenden Dinge tun?	**HANDLUNGSFÄHIGKEIT**<br>Haben oder hätten Sie, **unter Berücksichtigung Ihres Gesundheitszustands und Ihrer Funktionsfähigkeit**, **in den letzten vier Wochen Folgendes getan**?	**MÖGLICHKEIT ZU HANDELN**<br>Wenn Sie über **Ihr Leben im Allgemeinen** nachdenken, wie haben Sie **in den letzten vier Wochen** Ihre Möglichkeiten zur Durchführung der folgenden Dinge erlebt?	**AUSMASS DES HANDELNS**<br>Geben Sie an, in welchem Masse Sie die betreffenden Aktivitäten in den **letzten vier Wochen** ausgeführt haben.
**9**	Ich wollte Dinge tun, um meine sozialen Beziehungen aufrechtzuerhalten<br>○ 1. Sehr stark<br>○ 2. Ziemlich stark<br>○ 3. Zu einem gewissen Grad<br>○ 4. Nur ein bisschen<br>○ 5. Überhaupt nicht	Ich war oder wäre in der Lage gewesen Dinge zu tun, um meine sozialen Beziehungen aufrechtzuerhalten<br>○ 1. Ja, ohne Schwierigkeiten<br>○ 2. Ja, aber mit gewissen Schwierigkeiten<br>○ 3. Ja, aber mit grossen Schwierigkeiten<br>○ 4. Nicht ohne fremde Hilfe<br>○ 5. Nicht einmal mit Hilfe	Meine Möglichkeiten Dinge zu tun, um meine sozialen Beziehungen aufrechtzuerhalten waren<br>○ 1. Sehr gut<br>○ 2. Ziemlich gut<br>○ 3. Mittelmässig<br>○ 4. Eingeschränkt<br>○ 5. Es war nicht möglich	Ich habe Dinge getan, um meine sozialen Beziehungen aufrechtzuerhalten<br>○ 1. Sehr viel<br>○ 2. Ziemlich viel<br>○ 3. In gewissem Masse<br>○ 4. Nur ein bisschen<br>○ 5. Überhaupt nicht
**10**	Ich wollte Massnahmen ergreifen, um neue Bekanntschaften zu schliessen<br>○ 1. Sehr stark<br>○ 2. Ziemlich stark<br>○ 3. Zu einem gewissen Grad<br>○ 4. Nur ein bisschen<br>○ 5. Überhaupt nicht	Ich war oder wäre in der Lage gewesen Massnahmen zu ergreifen, um neue Bekanntschaften zu schliessen<br>○ 1. Ja, ohne Schwierigkeiten<br>○ 2. Ja, aber mit gewissen Schwierigkeiten<br>○ 3. Ja, aber mit grossen Schwierigkeiten<br>○ 4. Nicht ohne fremde Hilfe<br>○ 5. Nicht einmal mit Hilfe	Meine Möglichkeiten Massnahmen zu ergreifen, um neue Bekanntschaften zu schliessen waren<br>○ 1. Sehr gut<br>○ 2. Ziemlich gut<br>○ 3. Mittelmässig<br>○ 4. Eingeschränkt<br>○ 5. Es war nicht möglich	Ich habe Massnahmen ergriffen, um neue Bekanntschaften zu schliessen<br>○ 1. Sehr viel<br>○ 2. Ziemlich viel<br>○ 3. In gewissem Masse<br>○ 4. Nur ein bisschen<br>○ 5. Überhaupt nicht
**11**	Ich wollte Verantwortung übernehmen, um Angelegenheiten in Bezug auf mein eigenes Leben voranzutreiben<br>○ 1. Sehr stark<br>○ 2. Ziemlich stark<br>○ 3. Zu einem gewissen Grad<br>○ 4. Nur ein bisschen<br>○ 5. Überhaupt nicht	Ich war oder wäre in der Lage gewesen Verantwortung zu übernehmen, um Angelegenheiten in Bezug auf mein eigenes Leben voranzutreiben<br>○ 1. Ja, ohne Schwierigkeiten<br>○ 2. Ja, aber mit gewissen Schwierigkeiten<br>○ 3. Ja, aber mit grossen Schwierigkeiten<br>○ 4. Nicht ohne fremde Hilfe<br>○ 5. Nicht einmal mit Hilfe	Meine Möglichkeiten Verantwortung zu übernehmen, um Angelegenheiten in Bezug auf mein eigenes Leben voranzutreiben waren<br>○ 1. Sehr gut<br>○ 2. Ziemlich gut<br>○ 3. Mittelmässig<br>○ 4. Eingeschränkt<br>○ 5. Es war nicht möglich	Ich habe Verantwortung übernommen, um Angelegenheiten in Bezug auf mein eigenes Leben voranzutreiben<br>○ 1. Sehr viel<br>○ 2. Ziemlich viel<br>○ 3. In gewissem Masse<br>○ 4. Nur ein bisschen<br>○ 5. Überhaupt nicht
**12**	Ich wollte Verantwortung für die Förderung gesellschaftlicher Angelegenheiten oder Gemeindeangelegenheiten übernehmen<br>○ 1. Sehr stark<br>○ 2. Ziemlich stark<br>○ 3. Zu einem gewissen Grad<br>○ 4. Nur ein bisschen<br>○ 5. Überhaupt nicht	Ich war oder wäre in der Lage gewesen Verantwortung für die Förderung gesellschaftlicher Angelegenheiten oder Gemeindeangelegenheiten zu übernehmen<br>○ 1. Ja, ohne Schwierigkeiten<br>○ 2. Ja, aber mit gewissen Schwierigkeiten<br>○ 3. Ja, aber mit grossen Schwierigkeiten<br>○ 4. Nicht ohne fremde Hilfe<br>○ 5. Nicht einmal mit Hilfe	Meine Möglichkeiten Verantwortung für die Förderung gesellschaftlicher Angelegenheiten oder Gemeindeangelegenheiten zu übernehmen waren<br>○ 1. Sehr gut<br>○ 2. Ziemlich gut<br>○ 3. Mittelmässig<br>○ 4. Eingeschränkt<br>○ 5. Es war nicht möglich	Ich habe Verantwortung für die Förderung gesellschaftlicher Angelegenheiten oder Gemeindeangelegenheiten übernommen<br>○ 1. Sehr viel<br>○ 2. Ziemlich viel<br>○ 3. In gewissem Masse<br>○ 4. Nur ein bisschen<br>○ 5. Überhaupt nicht

Beantworten Sie jede Aussage (1-17)

	**WILLE ZU HANDELN**<br>Wie sehr wollten Sie **in den letzten vier Wochen** die folgenden Dinge tun?	**HANDLUNGSFÄHIGKEIT**<br>Haben oder hätten Sie, **unter Berücksichtigung Ihres Gesundheitszustands und Ihrer Funktionsfähigkeit**, **in den letzten vier Wochen Folgendes getan**?	**MÖGLICHKEIT ZU HANDELN**<br>Wenn Sie über **Ihr Leben im Allgemeinen** nachdenken, wie haben Sie **in den letzten vier Wochen** Ihre Möglichkeiten zur Durchführung der folgenden Dinge erlebt?	**AUSMASS DES HANDELNS**<br>Geben Sie an, wie oft oder wie viel Sie die betreffenden Aktivitäten in den **letzten vier Wochen** ausgeführt haben.
**13**	Ich wollte Dinge tun, um meine Tage interessanter oder reizvoller zu machen<br>○ 1. Sehr stark<br>○ 2. Ziemlich stark<br>○ 3. Zu einem gewissen Grad<br>○ 4. Nur ein bisschen<br>○ 5. Überhaupt nicht	Ich war oder wäre in der Lage gewesen meine Tage interessanter oder reizvoller zu machen<br>○ 1. Ja, ohne Schwierigkeiten<br>○ 2. Ja, aber mit gewissen Schwierigkeiten<br>○ 3. Ja, aber mit grossen Schwierigkeiten<br>○ 4. Nicht ohne fremde Hilfe<br>○ 5. Nicht einmal mit Hilfe	Meine Möglichkeiten meine Tage interessanter oder reizvoller zu machen waren<br>○ 1. Sehr gut<br>○ 2. Ziemlich gut<br>○ 3. Mittelmässig<br>○ 4. Eingeschränkt<br>○ 5. Es war nicht möglich	Ich habe meine Tage interessanter oder reizvoller gemacht<br>○ 1. Sehr viel<br>○ 2. Ziemlich viel<br>○ 3. Zu einem gewissen Grad<br>○ 4. Nur ein bisschen<br>○ 5. Überhaupt nicht
**14**	Ich wollte die Gemütlichkeit meines Hauses verbessern oder aufrechterhalten<br>○ 1. Sehr stark<br>○ 2. Ziemlich stark<br>○ 3. Zu einem gewissen Grad<br>○ 4. Nur ein bisschen<br>○ 5. Überhaupt nicht	Ich war oder wäre in der Lage gewesen die Gemütlichkeit meines Hauses zu verbessern oder aufrechtzuerhalten<br>○ 1. Ja, ohne Schwierigkeiten<br>○ 2. Ja, aber mit gewissen Schwierigkeiten<br>○ 3. Ja, aber mit grossen Schwierigkeiten<br>○ 4. Nicht ohne fremde Hilfe<br>○ 5. Nicht einmal mit Hilfe	Meine Möglichkeiten die Gemütlichkeit meines Hauses zu verbessern oder aufrechtzuerhalten waren<br>○ 1. Sehr gut<br>○ 2. Ziemlich gut<br>○ 3. Mittelmässig<br>○ 4. Eingeschränkt<br>○ 5. Es war nicht möglich	Ich habe die Gemütlichkeit meines Hauses verbessert oder aufrechterhalten<br>○ 1. Sehr viel<br>○ 2. Ziemlich viel<br>○ 3. Zu einem gewissen Grad<br>○ 4. Nur ein bisschen<br>○ 5. Überhaupt nicht
**15**	Ich wollte mich um mein äusseres Erscheinungsbild kümmern<br>○ 1. Sehr stark<br>○ 2. Ziemlich stark<br>○ 3. Zu einem gewissen Grad<br>○ 4. Nur ein bisschen<br>○ 5. Überhaupt nicht	Ich war oder wäre in der Lage gewesen mich um mein äusseres Erscheinungsbild zu kümmern<br>○ 1. Ja, ohne Schwierigkeiten<br>○ 2. Ja, aber mit gewissen Schwierigkeiten<br>○ 3. Ja, aber mit grossen Schwierigkeiten<br>○ 4. Nicht ohne fremde Hilfe<br>○ 5. Nicht einmal mit Hilfe	Meine Möglichkeiten mich um mein äusseres Erscheinungsbild zu kümmern waren<br>○ 1. Sehr gut<br>○ 2. Ziemlich gut<br>○ 3. Mittelmässig<br>○ 4. Eingeschränkt<br>○ 5. Es war nicht möglich	Ich habe mich um mein äusseres Erscheinungsbild gekümmert<br>○ 1. Sehr viel<br>○ 2. Ziemlich viel<br>○ 3. Zu einem gewissen Grad<br>○ 4. Nur ein bisschen<br>○ 5. Überhaupt nicht
**16**	Ich wollte mich darum kümmern, dass meine finanziellen Angelegenheiten in Ordnung sind<br>○ 1. Sehr stark<br>○ 2. Ziemlich stark<br>○ 3. Zu einem gewissen Grad<br>○ 4. Nur ein bisschen<br>○ 5. Überhaupt nicht	Ich war oder wäre in der Lage gewesen mich darum zu kümmern, dass meine finanziellen Angelegenheiten in Ordnung sind<br>○ 1. Ja, ohne Schwierigkeiten<br>○ 2. Ja, aber mit gewissen Schwierigkeiten<br>○ 3. Ja, aber mit grossen Schwierigkeiten<br>○ 4. Nicht ohne fremde Hilfe<br>○ 5. Nicht einmal mit Hilfe	Meine Möglichkeiten mich darum zu kümmern, dass meine finanziellen Angelegenheiten in Ordnung sind waren<br>○ 1. Sehr gut<br>○ 2. Ziemlich gut<br>○ 3. Mittelmässig<br>○ 4. Eingeschränkt<br>○ 5. Es war nicht möglich	Ich habe mich darum gekümmert, dass meine finanziellen Angelegenheiten in Ordnung sind<br>○ 1. Sehr viel<br>○ 2. Ziemlich viel<br>○ 3. Zu einem gewissen Grad<br>○ 4. Nur ein bisschen<br>○ 5. Überhaupt nicht
**17**	Ich wollte mich mit Dingen beschäftigen, die meinen Glauben oder meine Weltanschauung betreffen<br>○ 1. Sehr stark<br>○ 2. Ziemlich stark<br>○ 3. Zu einem gewissen Grad<br>○ 4. Nur ein bisschen<br>○ 5. Überhaupt nicht	Ich war oder wäre in der Lage gewesen mich mit Dingen zu beschäftigen, die meinen Glauben oder meine Weltanschauung betreffen<br>○ 1. Ja, ohne Schwierigkeiten<br>○ 2. Ja, aber mit gewissen Schwierigkeiten<br>○ 3. Ja, aber mit grossen Schwierigkeiten<br>○ 4. Nicht ohne fremde Hilfe<br>○ 5. Nicht einmal mit Hilfe	Meine Möglichkeiten mich mit Dingen zu beschäftigen, die meinen Glauben oder meine Weltanschauung betreffen waren<br>○ 1. Sehr gut<br>○ 2. Ziemlich gut<br>○ 3. Mittelmässig<br>○ 4. Eingeschränkt<br>○ 5. Es war nicht möglich	Ich habe mich mit Dingen beschäftigt, die meinen Glauben oder meine Weltanschauung betreffen<br>○ 1. Sehr viel<br>○ 2. Ziemlich viel<br>○ 3. Zu einem gewissen Grad<br>○ 4. Nur ein bisschen<br>○ 5. Überhaupt nicht
